# A novel factorial design implemented spectrofluorimetric determination of caffeic acid: individual and combined assays with curcumin

**DOI:** 10.1038/s41598-026-52002-y

**Published:** 2026-05-20

**Authors:** Walaa Nabil Abd-AlGhafar, Heba Elmansi, Marwa Elsbaey, Fathalla Belal, Mona Elsharkasy

**Affiliations:** 1 Pharmaceutical Analytical Chemistry Department, Faculty of Pharmacy, Mansoura University, P.O. Box 35516, Mansoura, Egypt; 2 Pharmacognosy Department, Faculty of Pharmacy, Mansoura University, P.O. Box 35516, Mansoura, Egypt

**Keywords:** Caffeic acid, Curcumin, Synchronous fluorimetry, Green tea, Human plasma, Biological techniques, Chemistry

## Abstract

In this research, we developed, for the first time, sensitive, selective, and reliable spectrofluorimetric methods for the quantitative analysis of caffeic acid (CAF) alone and in combination with curcumin (CUR). Two fluorimetric methods were established for the determination of CAF alone; a native fluorescence method (Method I) was optimized using 2^3^ factorial model, exploiting the compound’s intrinsic fluorescence properties. The excitation and emission wavelengths were set at λex/λem = [324/443] nm. For the simultaneous analysis of CAF and CUR, the synchronous fluorescence (SF) technique was employed (Method II). The constant wavelength difference was optimized at 100 nm, allowing the simultaneous quantification of both analytes in a single measurement without prior separation. Under optimized conditions, the native fluorescence method for CAF alone showed a linear range of 30.0–800.0 ng/mL with a lower detection limit of 9.44 ng/mL. The SF method for the mixture demonstrated linear ranges of 50.0-2000.0 ng/mL for CAF and 20.0-200.0 ng/mL for CUR, with LOD values of 15.40 and 5.94 ng/mL, respectively. Both methods were successfully validated according to ICH guidelines, demonstrating excellent accuracy, precision, and robustness. The proposed methods were applied to different matrices, including green tea (method I), synthetic mixtures, and spiked human plasma (methods I and II). The ecological impact of the proposed methods was investigated. They provide a simple, cost-effective, and green alternative to chromatographic techniques for the routine quality control of caffeic acid and curcumin.

**Supplementary Information:**

The online version contains supplementary material available at 10.1038/s41598-026-52002-y.

## Introduction

**Fig. 1:**
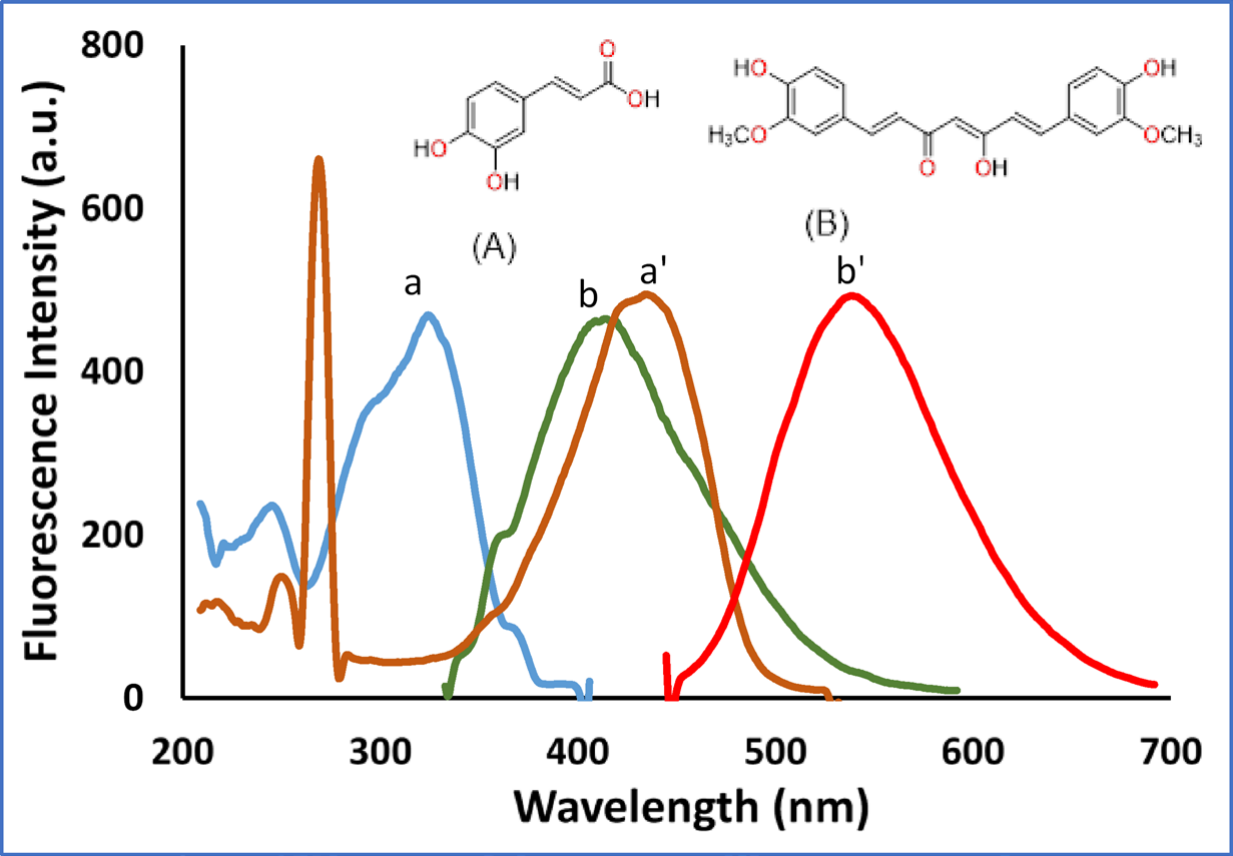
Excitation and emission fluorescence spectra of CAF (1000.0 ng/mL) (a, a´), and CUR (120.0 ng/mL) (b, b´) in ethanol (The insets: Chemical structure of (A) CAF and (B) CUR).

Caffeic acid (CA), chemically defined as trans-3-(3,4-dihydroxycinnamic acid), or (E)-3-(3,4-dihydroxyphenyl) prop-2-enoic acid (Fig. 1**inset**)^1^, is a widely distributed phenolic acid. Caffeic acid, a derivative of hydroxycinnamic acid, is structurally characterized as cinnamic acid with hydroxy substitutions at the 3 and 4 positions on its phenyl ring. While it can occur in both *cis* and *trans* isomeric configurations, the *trans* form is predominantly observed and more stable^1^. It is abundant in various fruits, vegetables, cereals, legumes, and beverages of plant origin such as wine, tea, and coffee, typically occurring in the form of esters with quinic acid^2,3^. Researchers have focused on CAF because of its promising health-promoting attributes, which are associated with a lowered risk of several major diseases, including cardiovascular diseases, cancer, and diabetes, as well as conditions linked to aging. The biological activity underpinning these benefits is believed to stem from its ability to protect against free radicals, mitigate inflammation, and inhibit viral infections^1–3^. Different techniques have been involved in CAF determination including; spectrophotometry^4,5^ and electrochemistry^6,7^. Additionally, several HPLC methods have been reported for analyzing CAF with other phenolic compounds^8–11^.

Curcumin (CUR), chemically defined as (1E,6E)-1,7-bis(4-hydroxy-3-methoxyphenyl) hepta-1,6-diene-3,5-dione (Fig. 1**inset**)^12^, is the principal bioactive compound in turmeric (Curcuma longa). Extensive research over the past two decades has highlighted its broad therapeutic potential, such as antioxidant, anti-inflammatory, and anticancer properties^13^. Notably, CUR has demonstrated considerable promise as a complementary agent to conventional anticancer treatments, owing to its capacity to modulate multiple signaling pathways involved in inflammation, cell proliferation, apoptosis, and drug resistance^14^. A number of analytical techniques have been developed for the detection and quantification of curcuminoids, including comparative assessments focused on turmeric rhizomes^15^. These include HPLC^16,17^, spectrofluorimetric^18,19^, and UPLC^20^ methods, reflecting continued interest in accurate CUR quantification.

Powerful synergies between nutraceuticals offer a promising avenue for addressing colon cancer, a malignancy with the second-highest mortality rate worldwide^21^. Polyphenol-rich extracts were reported to act synergistically with anticancer chemotherapeutics^22^. The efficacy of CUR and Caffeic Acid Phenethyl Ester against breast cancer has been evaluated and reported in preclinical studies^23^. Furthermore, CAF and CUR were found to possess anti-inflammatory properties and mitigated the cytotoxic effects of waterpipe tobacco smoke exposure^24^. Based on these findings, this study aims to develop new green and sensitive methods for quantification of CAF alone or in combination with CUR in pharmaceutical formulations, dietary supplements, and in vitro biological samples. The methods are intended for quality control applications and preclinical research, recognizing that both analytes undergo extensive phase II metabolism in vivo, which limits the clinical utility of parent compound quantification in human plasma after normal dietary or therapeutic doses^25–27^. However, for multiple analytes, synchronous fluorescence (SF) is used to resolve their overlapping spectra, enabling the simultaneous quantification of each component in a mixture without prior separation^28–31^.

Quality-by-Design (full factorial design FFD) is a statistics-based approach with numerous benefits to design, adapt, and validate the designed method^32,33^. This multivariate optimization takes less time, efforts, and resources than univariate procedures. Additionally, the implementation of experimental design permits a better improvement and comprehension of the performance of the established method by accurately identifying important variables and producing plots that illustrate its optimal performance and dependability^34^. Quality-by-design is attractive because they can classify the most important factors and examine their correlations^34^.

In this work, Method I is based on measuring the native fluorescence for CAF after getting the optimized conditions by using 2^3^ full factorial design. It was applied to assess CAF in green tea and human plasma samples. Method II depends on measuring both CAF and CUR simultaneously by SF. It was applied to synthetic mixtures and human plasma samples. A key objective is to evaluate and minimize the ecological impact of the proposed analytical procedures, aligning with the principles of green analytical chemistry^35^. The green credentials of the developed methods were rigorously evaluated using established assessment tools; Click Analytical Chemistry Index (CACI)^36^ and Analytical Green Star Area (AGSA)^37^.

## Experimental

### Instruments

Fluorometric analysis was conducted on an Agilent Technologies Cary Eclipse spectrofluorimeter, with data collection and processing managed by the integrated Cary Eclipse software. The instrumental parameters were set as follows:

- Method I: Fixed wavelength measurement at λ_ex = 324 nm and λ_em = 443 nm.
- Method II: Synchronous fluorescence scanning with a constant wavelength difference of Δλ = 100 nm.
- General Settings: A slit width of 5 nm and a smoothing factor of 20 were maintained for all experiments.

Solution pH was measured as needed using a calibrated Consort NV P-901 pH-Meter.

### Materials and solvents

All chemicals and reagents were of Analytical or HPLC grade. Authentic caffeic acid was obtained from Pharmacognosy Department, Faculty of Pharmacy, Mansoura University. NMR data is available at the supplemental file (**Figure S1**,** S2**) and **Table S1**, as compared to published data^38^. Curcumin (99.0%) was sourced from EMITCO Pharmaceuticals (Alexandria, Egypt). HPLC-grade organic solvents were procured from Fisher Scientific (Cairo, Egypt). Surfactants and stabilizers, including sodium dodecyl sulfate (SDS), sodium carboxymethyl cellulose (Na-CMC), Tween-80, Brij-35, and cetrimide, were obtained from Sigma-Aldrich (Seelze, Germany). Acetic acid, boric acid, phosphoric acid, and sodium hydroxide were obtained from El-Nasr Pharmaceutical Chemicals Co. (Cairo, Egypt).

Britton-Robinson buffer (BRB) solutions, covering a pH range of 2.1 to 12.0, were prepared and adjusted to the desired pH using a pH meter. Aqueous solutions were prepared with distilled water. Human plasma samples were kindly supplied by Mansoura University Hospitals (Mansoura, Egypt) and stored at − 80 °C until analysis. Green tea sachets were bought from local market, packed by El Youser Co. for Food Industries in 10th of Ramadan city, Egypt.

### Standard solutions

Individual stock solutions of CAF and CUR were prepared at a concentration of 200.0 µg/mL. This was achieved by accurately weighing 10.0 mg of each compound, transferring into separate 50-mL volumetric flasks, dissolving in ethanol, and diluting to the mark with the same solvent. The solutions were stable for at least one week. Working standard solutions of 10.0 µg/mL for each drug were generated by serial dilution with ethanol.

### General recommended procedures

#### Method I

Working solutions (30.0–800.0 ng/mL) were prepared by diluting appropriate aliquots of CAF stock solution into 10-mL volumetric flasks containing 0.8 mL BRB at pH 9.0, followed by dilution to volume with aqueous ethanol (50%) as a solvent. The fluorescence intensity (FI) was measured at λex/ λem = 324/443 nm. A calibration curve was constructed by plotting the blank-corrected FI values against the nominal CAF concentrations, and the regression equation was calculated.

#### Method II

Standard solutions of CAF (50.0–2000.0 ng/mL) and CUR (20.0–200.0 ng/mL) were prepared in 10-mL volumetric flasks by diluting appropriate aliquots to volume with ethanol. Synchronous fluorescence spectra were acquired at a constant wavelength difference (Δλ = 100 nm). The intensities were measured at 323 nm for CAF and 437 nm for CUR, with blank subtraction applied for baseline correction. Calibration curves were generated by plotting the corrected SF intensity against concentration, and the corresponding regression equations were calculated.

#### Application of method I to green tea

The green tea was finely powdered using a mortar, then 0.5 g of the powder (either non-spiked or spiked with CAF) were added to set of centrifuge tubes and completed to 10 mL with cold 95% methanol^39^. The tubes were sonicated for 5 min then centrifuged for 10 min at 3000 rpm. Then, 1 mL aliquot of the supernatant was transferred into 10-mL volumetric flask, the procedure under **“2.4.1. Method 1”** was conducted.

#### Application of method II to synthetic mixtures

Three different synthetic mixtures containing CAF and CUR within their analytical ranges were prepared in 10 mL volumetric flasks by combining appropriate aliquots of their working standard solutions. The samples were analyzed according to the recommended method **“2.4.2. Method II”**. The concentrations of each drug in the mixtures were determined by measuring the corrected synchronous fluorescence (SF) intensities and calculating the values using the pre-derived regression equations.

#### Application of the proposed methods to plasma samples

The applicability of the proposed methods to biological samples was evaluated using drug-spiked human plasma. Aliquots (1 mL) of plasma samples were spiked with varying concentrations of CAF alone (Method I) or CAF and CUR stock solutions to create synthetic mixtures (Method II). The solutions were properly mixed, and acetonitrile was used to precipitate plasma proteins, then adjusting the volume to 5.0 mL with acetonitrile. Following 3 min of vortex mixing, the mixtures were centrifuged at 4000 rpm for 30 min. The samples were processed according to the established procedures, alongside blank plasma for background correction. The concentrations were determined using the native **“Method I “**and synchronous **“Method II”** approaches, and the resulting data were used to construct calibration curves for quantitative analysis.

## Results and discussion

A primary goal in pharmaceutical development is to enhance therapeutic effectiveness while reducing toxicity. Evidence strongly supports the use of strategic drug combinations for improved efficacy through synergistic interactions. In this context, dietary bioactive compounds are significant contributors to health outcomes^40^. CAF and CUR are co-administered for a synergistic purpose. Their quantification is performed *via* spectrofluorimetry, a technique selected for its significant advantages, which include exceptional sensitivity, high selectivity, rapid analysis, cost-effectiveness, and low solvent consumption^28^. The proposed spectrofluorimetric methods are best suited for pharmaceutical quality control, dietary supplement analysis, in vitro studies, and preclinical pharmacokinetic investigations in animal models, where parent compound concentrations are higher. This approach is viable because the polyphenolic structures of both CAF and CUR exhibit intrinsic fluorescence, providing a basis for constructing highly sensitive analytical methods. Figure 1 shows the excitation and emission spectra for each of CAF and CUR in ethanol. The fluorescence of CAF was measured in a quantitative way and optimized to get optimum sensitivity (Method I). Due to the significant overlap of the spectra of both drugs, we utilized SF for their separation and quantitation simultaneously. Different variables were optimized to ensure maximum separation and sensitivity.

### Optimization of experimental conditions

#### For method I

Prior to employing a formal experimental design, preliminary screening tests were conducted to identify factors significantly influencing the FI of CAF. The evaluated factors included pH, buffer type, diluting solvent, and organized media. The screening revealed that buffer pH, type and volume were the most influential parameters, while the other factors had negligible effects. Upon optimization, it was found that CAF had high sensitivity in alkaline pH range from 9.0 to 10.0. Consequently, pH and buffer type and volume were selected as the independent variables for the design. Their experimental domains were established through initial testing: a pH range of 9 to 10, different buffers including Tris and BRB and a buffer volume range of 0.8 to 1.2 mL. As this volume range gave the highest RFI. Various solvents (water, ethanol, methanol, acetonitrile, and isopropanol) were evaluated for their impact on measured RFI value of buffered mixture of CAF. Ethanol gave high sensitivity with attractive greenness (**Figure S3**). Various buffers involving 0.04 M BRB, 0.2 M borate buffer, and 0.1 M tris buffer were tried to get the optimum RFI. Upon dilution of the BRB and borate buffer with ethanol, precipitation occurred. So, it was concluded using factorial design that dilution of BRB pH of 9.0 with 50% ethanol gave the optimum RFI.

**Fig. 2:**
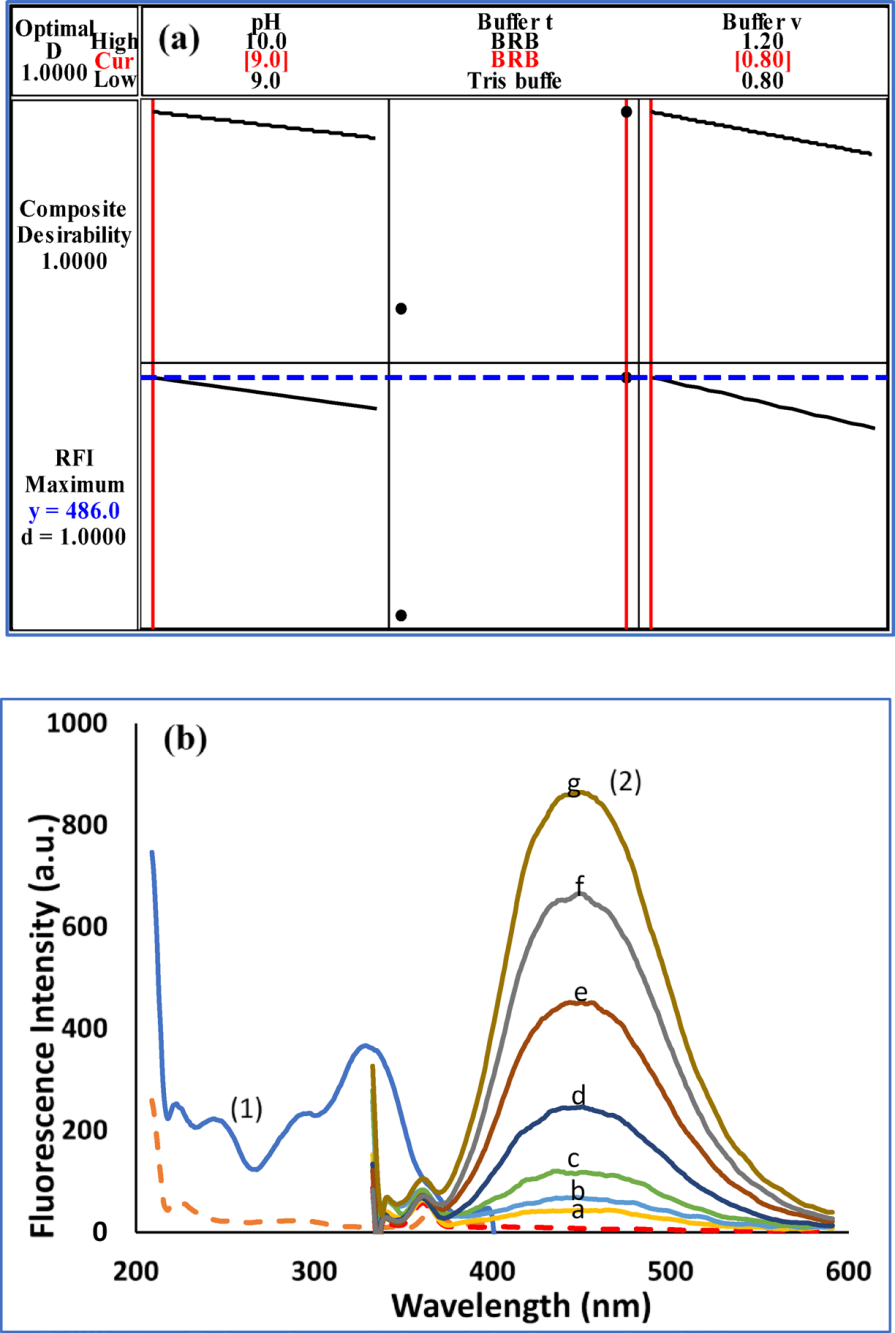
(**a**) FFD optimization plot and (**b**) Fluorescence spectra of CAF (1) excitation and (2) emission (a-g; 30.0, 50.0, 100.0, 200.0, 400.0, 600.0, and 800.0 ng/mL) in 0.8 mL of BRB (pH = 9.0) using water and ethanol (1:1) as a solvent.

A 2^3^ FFD, comprising four experiments, was subsequently implemented to determine the optimal factor settings for maximizing the response (Table 1). The corrected fluorescence intensity for each experiment was recorded and analyzed using Minitab^®^ software. The response optimizer function was employed to maximize both individual (d) and composite (D) desirability values (**Table S2**). The resulting optimization plot (Fig. 2a) illustrates the effect of each factor on the response and was used to identify the optimal experimental conditions. These validated optimum conditions were then applied for all subsequent analyses. Figure 2b shows the fluorescence spectra of increasing concentrations of CAF in 0.8 mL of BRB (pH = 9.0) using water and ethanol (1:1) as a solvent.

**Table 1:** 2^3^ experimental factorial designs and their dependent response for method I assay of CAF.

Design order		Experimental factorial design			Dependent response
Std order	**Run order**	**pH (A)**	**Buffer type (B)**	**Buffer volume (mL) (C)**	**RFI**
2	1	10.0	Tris buffer	0.8	380
6	2	10.0	Tris buffer	1.2	344
3	3	9.0	BRB	0.8	486
8	4	10.0	BRB	1.2	449
1	5	9.0	Tris buffer	0.8	319
4	6	10.0	BRB	0.8	464
5	7	9.0	Tris buffer	1.2	283
7	8	9.0	BRB	1.2	450

Based on Pareto Chart (**Figure S4a**), main effects plots (**Figure S4b**), and normal plot (**Figure S4c**), buffer type (B) has the greatest significant impact on RFI. BRB volume (C) has negative impact on it. The interaction plots (**Figure S4d**) point out that BRB volume exhibits negative impact when it interacts with other factors either at high or low levels.

#### For method II

##### Choice of wavelength difference (Δλ)

The Δλ value is critical for synchronous fluorescence sensitivity, resolution, and spectral shape. The optimal Δλ for analyzing CAF and CUR was determined by scanning a wide range (20–160 nm). A Δλ of 100 nm was selected as it produced sharp, well-resolved peaks with maximum intensity and minimal overlap (**Figure S5**). Any deviation from this value decreased both resolution and signal strength.

##### Effect of diluting solvent

Different solvents were investigated. It was concluded that ethanol is the optimum choice in the proposed method (**Figure S3**).

##### Effect of pH

The fluorescence intensity was evaluated across the pH range of 2.1–12.0 using acetate, borate, and phosphate buffers. In spite of the enhancement of the fluorescence signal of CAF in pH range of 8.0–11.0, these conditions greatly drop that of CUR (**Figure S6**), so the method was executed without pH adjustment, using pure ethanol as the solvent. The opposite pH-dependent behavior of CAF and CUR is explained by their distinct molecular structures (Fig. 1). CAF contains ionizable phenolic and carboxylic groups; deprotonation at alkaline pH creates an extended conjugated system with intramolecular charge transfer (ICT), enhancing fluorescence. CUR, however, contains a β-diketone enol moiety that is prone to deprotonation and subsequent hydrolytic degradation at alkaline pH *via* retro-aldol cleavage, destroying its fluorescent chromophore^41^.

##### Effect of surfactant

A systematic study was conducted to assess the ability of organized media to act as fluorescence-enhancing agents for CAF and CUR. Several surfactants (Tween-80, cetrimide, SDS, Brij, Kolliphor RH 40) and macromolecules (CMC, β-CD) were tested at concentrations exceeding their critical micelle concentrations. The results indicated that none of these media yielded a substantial increase in the fluorescence intensity for either analyte (**Figure S7**). Therefore, they were excluded from the final optimized analytical method.

The lack of fluorescence enhancement by surfactants for both CAF and CUR can be attributed to the molecular structures and inherent photophysical properties of these two drugs. The lack of enhancement for CAF can be attributed to its anionic nature at the experimental pH (pKa ~ 4.2 and ~ 8.5–9.5)^42^. At alkaline pH (Method I, pH 9.0) or in neutral ethanol (Method II), CAF exists predominantly as a negatively charged species. This anionic form experiences electrostatic repulsion from anionic surfactants (SDS) and shows weak partitioning into non-ionic or cationic micelles due to its high water solubility. Moreover, the inherent rigidity of CAF’s conjugated cinnamic acid backbone, stabilized by intramolecular hydrogen bonding, means that additional conformational restriction from micellar incorporation does not significantly reduce non-radiative decay pathways. For CUR, the absence of surfactant enhancement is more complex. CUR is known to be photolabile and undergoes rapid degradation at alkaline pH, where many surfactants are optimally effective.

**Fig. 3:**
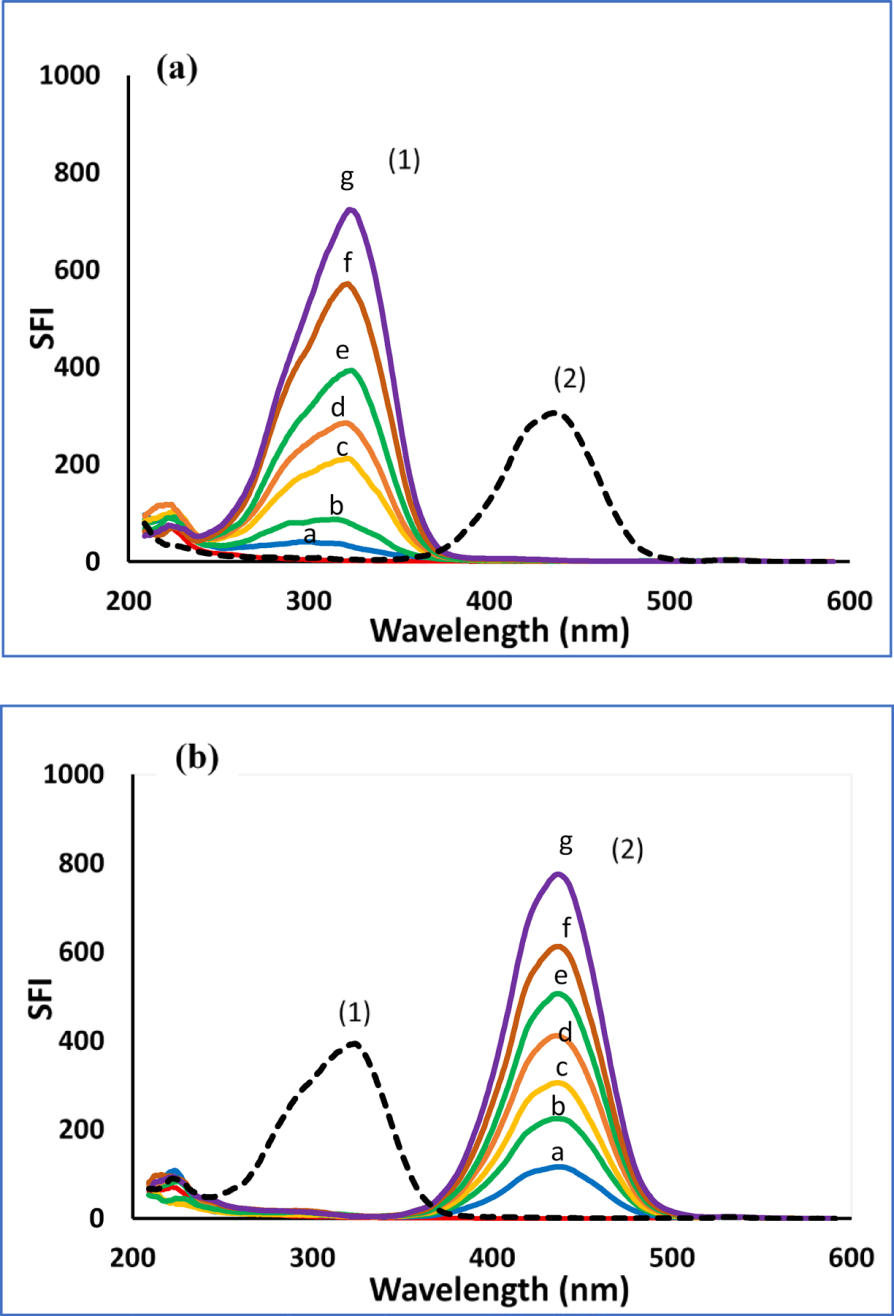
(**a**) Synchronous fluorescence spectra of (1) CAF (a-g; 50.0, 200.0, 500.0, 700.0, 1000.0, 1500.0, and 2000.0 ng/mL) and (2) CUR (70.0 ng/mL) and (**b**) Synchronous fluorescence spectra of (1) CAF (1000.0 ng/mL) and (2) CUR (a-g; 20.0, 50.0, 70.0, 100.0, 120.0, 150.0, and 200.0 ng/mL).

The application of the optimized parameters enabled the accurate, simultaneous quantification of each drug within their respective linear ranges. As shown in Figs. 3a **and b**, the assay demonstrated high selectivity with no detectable cross-interference between the analytes.

### Validation parameters

In accordance with International Council for Harmonization (ICH) guidelines^43^, the methods were validated by assessing key parameters including linearity, range, accuracy, precision, robustness, limits of detection (LOD) and quantitation (LOQ), and selectivity.

Calibration curves for both methods were constructed and demonstrated excellent linearity, as indicated by their high correlation coefficients (r). For Method I, a native spectrofluorimetric assay for CAF, the relative fluorescence intensity (RFI) was plotted against concentrations ranging from 30.0 to 800.0 ng/mL. For Method II, the measured SFI was plotted against concentration, showing linear ranges of 50.0–2000.0 ng/mL for CAF and 20.0–200.0 ng/mL for CUR. The corresponding regression equations and key statistical parameters for both methods are comprehensively summarized in Table 2.

**Table 2:** Analytical data for the studied methods.

Validation parameter	Method I	Method II	
	CAF	CAF	CUR
Wavelength (nm)	443	323	437
Linearity range (ng/mL)	30.0-800.0	50.0-2000.0	20.0-200.0
Number of experiments (n)	7	7	7
Intercept (a)	17.350	22.480	43.927
Slope (b)	1.065	0.353	3.711
Correlation coefficient (r)	0.9999	0.9999	0.9994
Standard deviation of residuals (S_y/x_)	5.35	2.65	8.64
Standard deviation of intercept (S_a_)	3.05	1.64	6.67
Standard deviation of slope (S_b_)	7.0 × 10^− 3^	1.5 × 10^− 3^	5.7 × 10^− 2^
%Relative standard deviation (%RSD)	1.46	0.83	1.66
%Error	0.55	0.32	0.63
LOD (ng/mL)	9.44	15.40	5.94
LOQ (ng/mL)	28.60	46.66	17.98

The accuracy of the proposed methods was confirmed through a comparative study with established reference methods^4,18^. Statistical analysis using Student’s t-test and the variance ratio *F*-test^44^ revealed no significant differences in accuracy or precision between the approaches (Table 3), validating the new methods.

**Table 3:** Estimation of CAF and CUR in raw materials.

	Method I			Method II					
	CAF at (443 nm)			CAF at (323 nm)			CUR at (437 nm)		
Parameter	Concentration		%found^a^	Concentration		%found^a^	Concentration		%found^a^
	Taken (ng/mL)	Found (ng/mL)		Taken (ng/mL)	Found (ng/mL)		Taken (ng/mL)	Found (ng/mL)	
	30.0	29.211	97.37	50.0	49.869	99.74	20.0	19.638	98.19
	50.0	49.225	98.45	200.0	197.891	98.95	50.0	49.029	98.06
	100.0	98.514	98.51	500.0	498.163	99.63	70.0	70.307	100.44
	200.0	198.845	99.42	700.0	709.801	101.40	100.0	99.039	99.04
	400.0	406.036	101.51	1000.0	991.252	99.13	120.0	122.159	101.80
	600.0	605.586	100.93	1500.0	1506.88	100.46	150.0	152.868	101.91
	800.0	793.310	99.16	2000.0	1995.93	99.80	200.0	197.189	98.59
X̅ ± SD	99.34 ± 1.45			99.87 ± 0.83			99.72 ± 1.66		
%RSD	1.46			0.83			1.66		
	Comparison method [4] (*n* = 3)			Comparison method [4] (*n* = 3)			Comparison method [18] (*n* = 3)		
X̅ ± SD	100.08 ± 1.16			100.08 ± 1.16			99.85 ± 0.80		
*t* ^b^	0.86 (2.31)			0.28 (2.31)			0.17 (2.31)		
F^b^	1.56 (5.14)			1.95 (5.14)			4.31 (5.14)		

^a^Average of three replicate determinations.

^b^ The theoretical t and F values (*P* = 0.05) are between parentheses^44^.

Furthermore, the methods’ precision was rigorously evaluated. The intra-day and inter-day precision, assessed by analyzing three different concentrations across the established linear ranges, yielded low % relative standard deviation (%RSD) and percentage error (%Er) values. These results, presented in Table 4, confirm the methods’ excellent reproducibility and reliability for routine analysis.

**Table 4:** Intra-day and inter-day precision data for the studied drugs using the proposed methods.

Method I					
CAF					
Concentration (ng/mL)		**Intraday precision**		**Interday precision**	
		X̅ ± SD	%RSD	X̅ ± SD	%RSD
	100.0	98.51 ± 0.24	0.24	98.35 ± 1.89	1.92
	200.0	101.0 ± 1.37	1.36	100.17 ± 1.77	1.76
	400.0	101.63 ± 0.16	0.16	100.92 ± 1.95	1.93
Method II					
Concentration (ng/mL)					
CAF	200.0	98.25 ± 0.62	0.63	98.44 ± 0.61	0.62
	700.0	99.32 ± 1.84	1.86	97.94 ± 0.66	0.67
	1000.0	99.43 ± 1.31	1.31	99.32 ± 0.62	0.63
CUR	50.0	100.09 ± 1.93	1.93	98.41 ± 1.64	1.66
	100.0	99.23 ± 1.49	1.50	98.85 ± 1.80	1.82
	150.0	102.43 ± 0.53	0.51	100.25 ± 2.07	2.06

The detection and quantitation limits were mathematically determined following the ICH Q2(R1) guidelines [43]. The analysis revealed detection limits of 9.44 ng/mL for CAF in Method I and 15.40 and 5.94 ng/mL for CAF and CUR in Method II, while the quantitation limits were 28.60 ng/mL for CAF in Method I and 46.66 and 17.98 ng/mL, respectively for CAF and CUR in Method II. These findings highlight the exceptional sensitivity of the synchronous spectrofluorimetric method, making it suitable for measuring these drugs in human plasma samples (Table 2).

The robustness of Method I was evaluated by investigating the impact of variations in two key parameters: the pH (8.8–9.2) and the volume (0.6-1.0) of the BRB buffer. Deliberate, small changes were introduced to these factors to assess their influence on the analytical results. The method demonstrated satisfactory robustness, as these minor modifications did not significantly affect the outcome, confirming its reliability for routine analysis under normal variations in experimental conditions (Table 5).

**Table 5:** Robustness study of the studied method I for the analysis of CAF (400.0 ng/mL) in raw material.

Parameter	CAF
pH	%Recovery^a^
8.8	101.50
9.0	101.51
9.2	102.51
SD	0.58
BRB volume (mL)	
0.6	101.03
0.8	101.51
1.0	101.81
SD	0.39

Method I exhibited excellent selectivity for CAF. The method was examined with common pharmaceutical excipients for potential interferents, including anions, metal ions, sugars, and talc powder. As shown in **Table S3**, recovery percentages for CAF (400.0 ng/mL) consistently ranged from 96.41% to 102.51% (± SD ≤ 1.50). These results, showing minimal deviation and low variability, confirm the method’s selectivity.

### Application to synthetic mixtures

**Fig. 4:**
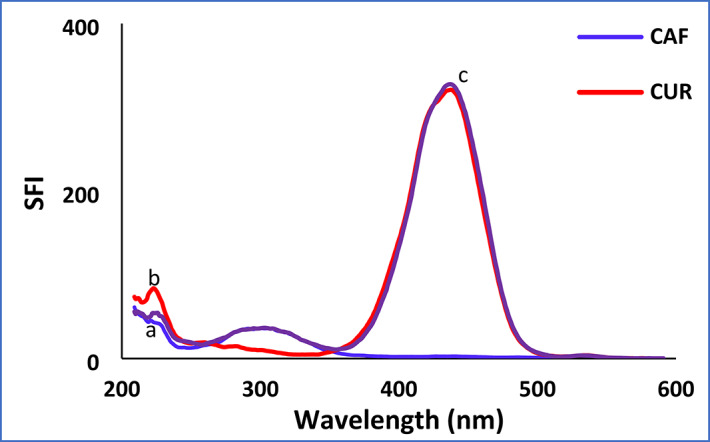
Synchronous fluorescence spectra of: (**a**) CAF (70.0 ng/mL), (**b**) CUR (70.0 ng/mL), and (**c**) synthetic mixture of both.

To evaluate its applicability, the proposed spectrofluorimetric approach (Method II) was applied to the analysis of synthetic mixtures containing CAF and CUR in different proportions (70.0:50.0, 100.0:70.0, 200.0:100.0 ng/mL, respectively). The results demonstrated that each drug could be accurately and precisely quantified in presence of each other (Fig. 4). This was further supported by the high percentage recoveries calculated for all mixture ratios, which are comprehensively listed in Table 6.

**Table 6:** Assay results for the estimation of CAF and CUR in synthetic mixtures using method II.

Parameter	Concentration taken (ng/mL)		Concentration found (ng/mL)		%found	
	**CAF**	**CUR**	**CAF**	**CUR**	**CAF**	**CUR**
	70.0	50.0	69.559	48.125	99.37	96.25
	100.0	70.0	97.928	70.790	97.93	101.13
	200.0	100.0	200.056	101.351	100.03	101.35
X̅ ± SD					99.11 ± 1.07	99.58 ± 2.88
%RSD					1.08	2.90
%Error					0.63	1.67

### Application to spiked human plasma samples

**Fig. 5:**
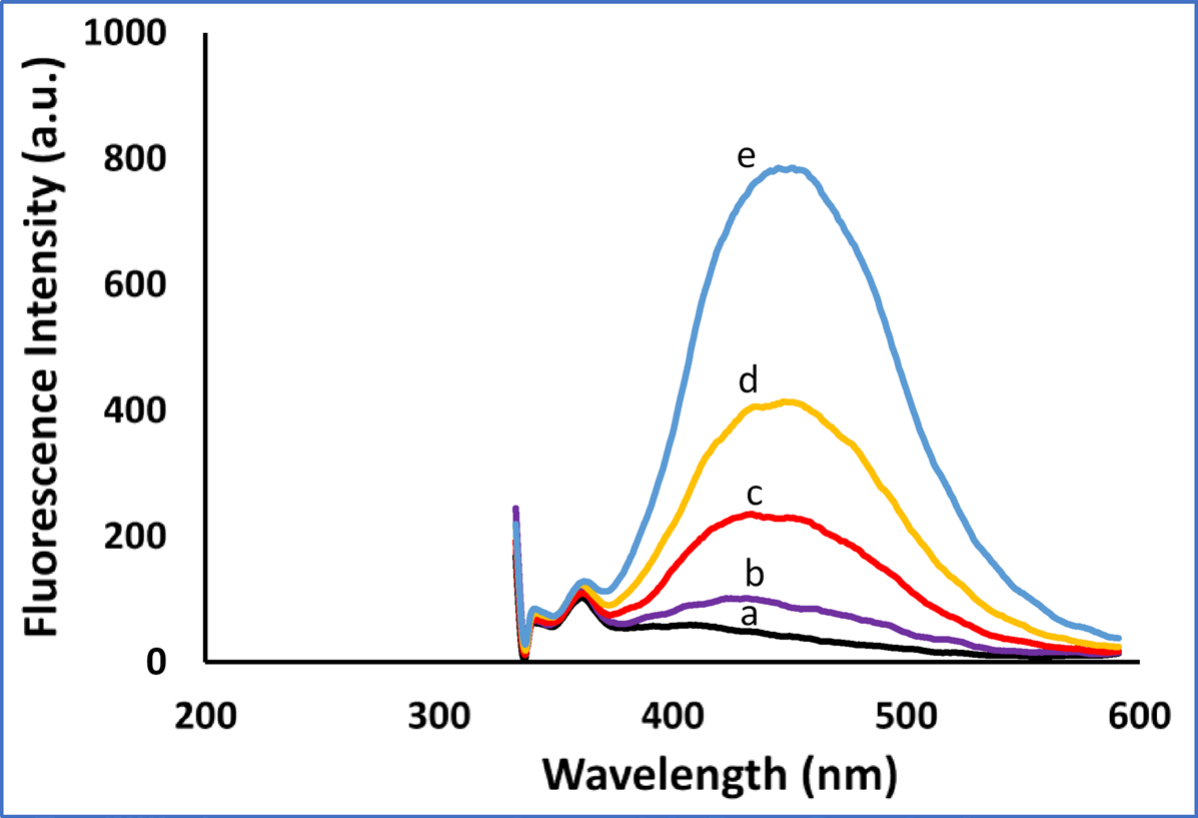
Application of CAF in spiked human plasma where: (a) Blank (Plasma). (b-e concentration of drugs spiked in plasma samples (60.0, 200.0, 400.0, and 800.0 ng/mL).

**Fig. 6:**
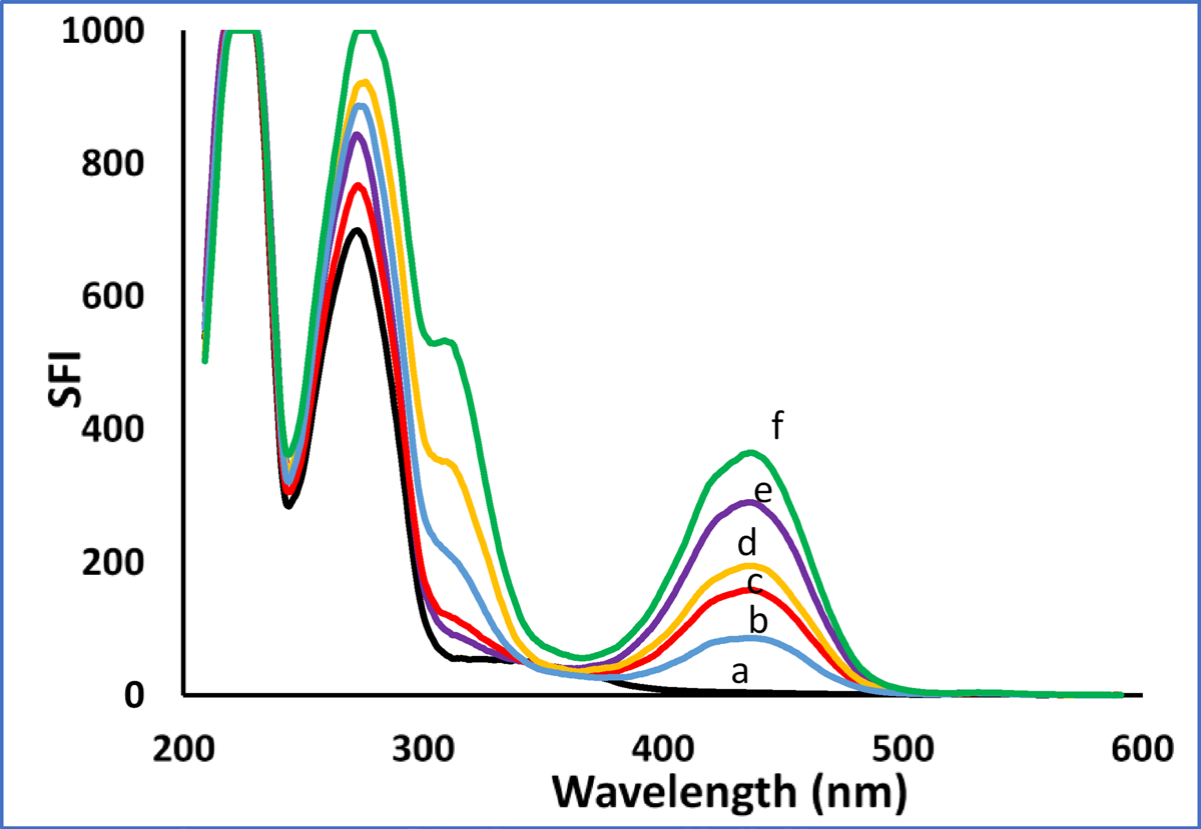
Application of drugs in spiked human plasma where: (a) Blank (Plasma). (b-f; concentration of CAF and CUR in spiked plasma samples (100.0 + 100.0 ng/mL), (200.0 + 50.0 ng/mL), (1000.0 + 70.0 ng/mL), (500.0 + 30.0 ng/mL), and (1500.0 + 120.0 ng/mL) respectively.

The clinical applicability of the two spectrofluorimetric methods was validated by their excellent performance in the analysis of spiked human plasma samples for both CAF and CUR. The methods are sufficiently sensitive to detect therapeutically relevant concentrations of CAF and CUR [45,46]. CAF reaches a C_max_ of approximately 880 ng/mL in human plasma after dietary intake [45], which lies well within the linear range of Method I (30.0–800.0 ng/mL). For CUR, despite its poor oral bioavailability (C_max_ ~ 60 ng/mL after a 500 mg dose/kg [46]), the LOQ of Method II (17.98 ng/mL) can reach the analysis of trace-levels in plasma in pharmacokinetic studies, pharmaceutical formulations, and bioavailable curcumin preparations [18,19]. Thus, the proposed methods offer practical utility across a range of applications, from dietary monitoring to pharmaceutical quality control. For method I (Fig. 5), different concentrations were analyzed; 60.0, 200.0, 400.0 and 800.0 ng/mL. For method II (Fig. 6), different ratios for both CAF and CUR were tested; (100.0 + 100.0 ng/mL), (200.0 + 50.0 ng/mL), (1000.0 + 70.0 ng/mL), (500.0 + 30.0 ng/mL), and (1500.0 + 120.0 ng/mL), for CAF and CUR, respectively. The acceptable percentage recoveries and low %RSD values for a range of spiked concentrations (Table 7) indicate high accuracy and precision, establishing the methods as reliable tools for therapeutic drug monitoring.

**Table 7:** Application of the developed approaches in green tea and spiked human plasma.

Parameter	Method I			
	Spiked (mg/100 g)		Found (mg/100 g)	%Recovery
Green tea	4		3.874	96.85
	12		12.491	104.09
	28		27.699	98.93
X̅ ± SD				99.96 ± 3.73
%RSD				3.73
	Concentration taken (ng/mL)		Concentration found (ng/mL)	%Recovery
Human plasma	60.0		61.356	102.26
	200.0		199.431	99.72
	400.0		398.222	99.56
	800.0		800.976	100.12
X̅ ± SD				100.42 ± 1.25
%RSD				1.25
r				0.9999
CAF	Method II in human plasma			
	100.0		105.234	105.23
	200.0		202.047	101.02
	500.0		505.117	101.02
	1000.0		968.933	96.89
	1500.0		1518.530	101.24
X̅ ± SD				101.08 ± 2.95
%RSD				2.92
r				0.9995
CUR	30.0		30.896	102.99
	50.0		51.677	103.35
	70.0		67.326	96.18
	100.0		97.420	97.42
	120.0		122.794	102.33
X̅ ± SD				100.45 ± 3.38
%RSD				3.37
r				0.9979

### Application for green tea

The developed method I successfully applied to the quantification of CAF in commercially available green tea packets. Samples were prepared using a simple extraction protocol. The study revealed that CAF content in the green tea was 22.72 mg/100 g. This value was comparable to that reported by Kochman et al. [47]. The standard addition experiment was later conducted. The results demonstrated excellent recovery with minimal matrix interference, confirming the method’s suitability for complex natural products (Table 7).

### Practicality and greenness assessment

The practical utility of the synchronous spectrofluorimetric method was assessed *via* the CACI [36], an established metric for evaluating methodological feasibility. The a3pproach attained a favorable CACI score of 84 (**Figure S8**), reflecting its cost efficiency, operational simplicity, and strong potential for routine application. Environmental impact was further scrutinized using the AGSA metric [37], aligned with the 12 Principles of Green Analytical Chemistry. The method achieved an outstanding AGSA score of 83.33%, categorizing it as “Excellent Green”. Notable green attributes include the use of ethanol; a safe, renewable solvent in place of hazardous organic reagents, and the inherent efficiency of synchronous fluorescence, which allows simultaneous quantification of CAF and CUR without energy-intensive chromatographic separation, thereby minimizing analysis time, waste generation, and overall resource use. Together, the strong CACI and AGSA scores underscore that this approach successfully integrates practical applicability with exceptional environmental and analytical performance, positioning it as a promising framework for pharmaceutical quality control and foundational step toward sustainable bioanalytical applications.

## Conclusion

The combination of CUR with CAF represents a promising multimodal strategy to enhance therapeutic efficacy. CUR may complement the action of CAF by targeting synergistic pathways. In this study, two novel, sensitive, and selective spectrofluorimetric methods were developed and validated for the quantification of CAF and the simultaneous determination of CAF and CUR. The first approach employed direct spectrofluorimetry for highly sensitive and precise measurement of CAF in aqueous ethanol (50%) and BRB at pH 9.0. The second method utilized synchronous spectrofluorimetry to resolve the overlapping spectra of CAF and CUR in ethanol without requiring physical separation, offering a rapid and efficient means for their concurrent analysis. The approach was optimized utilizing two-level full factorial design to save time, effort, and resources. Both methods exhibited excellent linearity, accuracy, and precision in compliance with ICH guidelines.

These methods provide rapid, cost-effective, and environmentally sustainable alternatives to conventional chromatographic methods, making them highly suitable for pharmaceutical quality control and therapeutic drug monitoring. Their successful application in different matrices was demonstrated, underscoring their practical utility. Furthermore, the green credentials of both methods were rigorously evaluated using two modern assessment tools, confirming their alignment with green analytical chemistry principles. The combination of analytical performance, operational simplicity, and ecological sustainability makes these spectrofluorimetric methods highly advantageous for routine use in clinical and pharmaceutical settings.

## Electronic Supplementary Material

Below is the link to the electronic supplementary material.

Supplementary Material 1

## Data Availability

The datasets used and/or analyzed during the current study available from the corresponding author on reasonable request.
