# Supplementary material for: A novel factorial design implemented spectrofluorimetric determination of caffeic acid: individual and combined assays with curcumin
Source: Sci Rep. 2026 May 20;16:15717. doi: 10.1038/s41598-026-52002-y (PMC13190843; doi:10.1038/s41598-026-52002-y)
Supplement: Supplementary file 1 — Supplementary Material 1 [file 41598_2026_52002_MOESM1_ESM.docx]

**Fig. S1.** ^1^H NMR spectrum of authentic caffeic acid (400 MHz, CD_3_OD).

**Fig. S2.** APT spectrum of authentic caffeic acid (100 MHz, CD_3_OD).

**Fig. S3:** Effect of diluting solvent on RSFI of CAF (1000.0 ng/mL) and CUR (120.0 ng/mL).

**Fig. S4:** 2^3^ full factorial design (a) Pareto chart of the effects on the RFI at alpha = 0.05, (b) main effect plot for RFI by data means type, (c) Normal plot, and (d) interaction plots for RFI by data means type.

**Fig.S5:** Synchronous Fluorescence spectra of (a) CAF (1500.0 ng/mL) and (b) CUR (120.0 ng/mL) at different Δλ settings.

**Fig. S6:** Effect of buffer solutions of different pH on RSFI of CAF (1000.0 ng/mL) and CUR (120.0 ng/mL).

**Fig. S7:** Effect of different surfactant on RSFI of CAF (1000.0 ng/mL) and CUR (120.0 ng/mL).

**Fig.S8**: Investigation of greenness and practicality using CACI and AGREE tools.

**Figure S1.**

**Figure S2.**

**Figure S3**

**(b)**

**(a)**

**(d)**

**(c)**

**Figure S4**

**Figure S5**

**Figure S6**

**Figure S7**

**
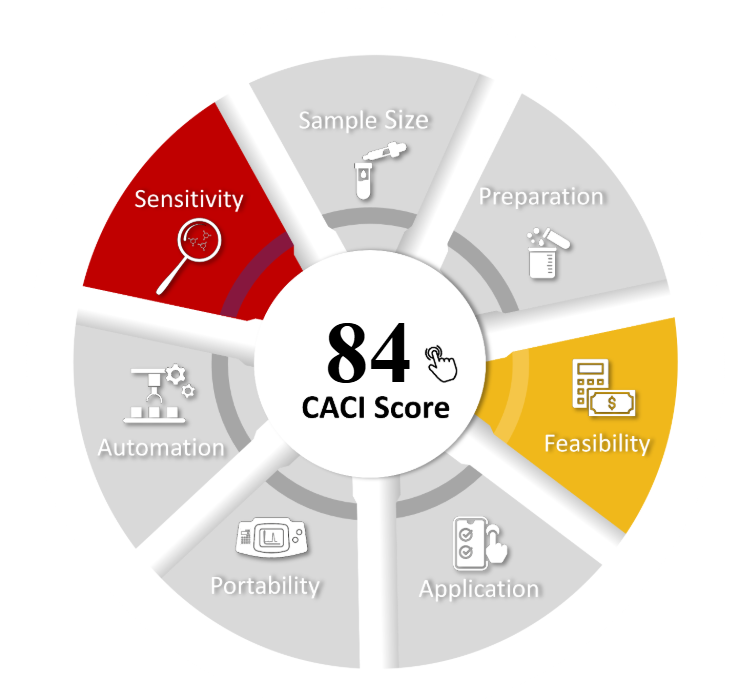
** **
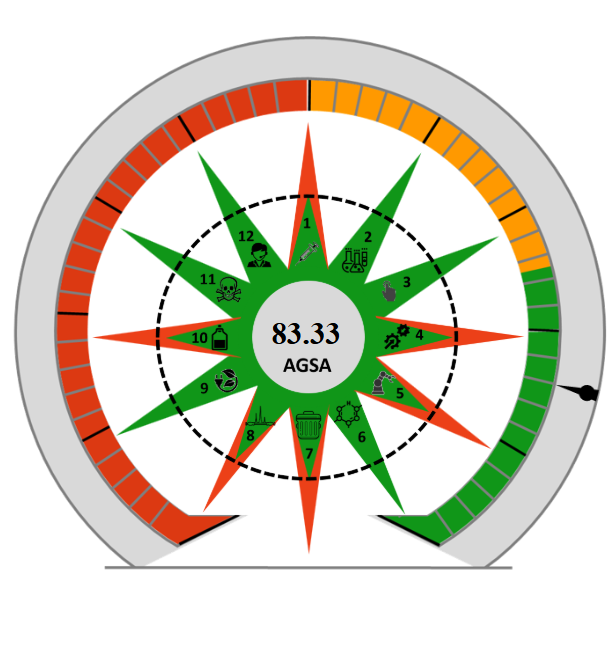
**

**Figure S8**

**Table S1**. NMR data of authentic caffeic acid versus published data.

| Position | **Authentic caffeic acid^a^** | | **Published data of caffeic acid ^b^** [38] | |
| --- | --- | --- | --- | --- |
|  | δ_C_ | δ_H,_ mult (*J* in Hz) | δ_C_ | δ_H,_ mult (*J* in Hz) |
| 1 | 128.2 | - | 127.7 | - |
| 2 | 115.5 | 6.94, d (1.6) | 115.1 | 7.04, d (2.0) |
| 3 | 147.1 | - | 146.8 | - |
| 4 | 149.9 | - | 149.4 | - |
| 5 | 116.9 | 6.68, d (8.2) | 116.5 | 6.75, d (8.0) |
| 6 | 123.3 | 6.83, dd (8.2, 1.6) | 122.8 | 6.93, dd (8.0, 2.0) |
| 7 | 147.4 | 7.43, d (15.9) | 147.1 | 7.57, d (16.0) |
| 8 | 116.0 | 6.12, d (15.9) | 115.5 | 6.31, d (16.0) |
| 9 | 171.6 | - | 171.1 | - |

^a^NMR recorded in CD_3_OD at 400 MHz for 1H and 100 MHz for 13C NMR

^b^NMR recorded in CD_3_OD at 500 MHz for 1H and 125 MHz for 13C NMR

**Table S2**

Response optimization of 2^3^ full factorial design for method I assay of CAF.

|  | Parameters | | | | | | Optimum conditions:  pH = 9.0,  Buffer type: Tris buffer, BRB volume = 0.8 mL | |
| --- | --- | --- | --- | --- | --- | --- | --- | --- |
|  |  |  |  |  |  |  | **Composite desirability (D) = 1.0** | |
| Responses | Goal | Lower | Target | Upper | Weight | Import | Predicted responses | Individual desirability (d) |
| RFI | Maximize | 283 | 486 | - | 1.0 | 1.0 | 486 | 1.0 |

**Table S3**

Evaluation of the selectivity of the studied method I in presence of common excipients.

| Excipients  each concentration 10 mM | %found ± SD |
| --- | --- |
|  | CAF  (400.0 ng/mL) |
| Cl^-^ | 101.80 ± 0.24 |
| S^2-^ | 99.22 ± 0.40 |
| S_2_O_3_^2-^ | 103.21 ± 0.89 |
| Na^+^ | 102.04 ± 0.62 |
| Cd^2+^ | 99.69 ± 0.80 |
| Co^2+^ | 102.51 ± 0.29 |
| Ni^2+^ | 101.97 ± 0.24 |
| Ba^2+^ | 101.81 ± 1.28 |
| Mg^2+^ | 103.68 ± 1.50 |
| Fe^3+^ | 96.41 ± 0.84 |
| Glucose | 101.83 ± 0.95 |
| Lactose | 101.81 ± 1.33 |
| Talc | 98.38 ± 0.40 |
